# Understanding maternal Ethnomedical Folklore in Central Uganda: a cross-sectional study of herbal remedies for managing Postpartum hemorrhage, inducing uterine contractions and abortion in Najjembe sub-county, Buikwe district

**DOI:** 10.1186/s12905-024-03205-w

**Published:** 2024-06-17

**Authors:** Alice Nabatanzi, Abdul Walusansa, Joanita Nangobi, Doreen Apio Natasha

**Affiliations:** 1 Department of Plant Sciences, Microbiology, and Biotechnology, College of Natural Sciences, Makerere University, Kampala, Uganda; 2 Department of Medical Microbiology, Faculty of Health Sciences, Habib Medical School, Islamic University, Mbale, Uganda; 3 Department of Pharmacy, College of Health Sciences, Makerere University, Kampala, Uganda

**Keywords:** Pregnancy, Uterotonics, Abortion, Postpartum haemmorhage, Medicinal plants, Buikwe

## Abstract

**Abstract:**

Pregnant women in rural Uganda largely rely on medicinal plants for inducing labor, treating postpartum hemorrhage (PPH), and inducing abortion. 90% of the women in both rural and urban Uganda use plants to manage pregnancy symptoms like constipation, heartburn, morning sickness, body aches, nausea, and vomiting. After delivery women continue using plants to manage postpartum complications and for infant care especially herbal baths. This study documented how ethnomedical folklore has been used to aid childbirth, manage postpartum hemorrhage, and induce abortion.

**Methods:**

A cross-sectional ethnobotanical survey was conducted from May – December 2023 in Najjemebe sub-county, Buikwe district. 206 respondents from 12 villages were selected using snowball sampling. Key informants included Traditional Birth Attendants (TBAs) and herbalists. Data was collected using semi-structured questionnaires and focus group discussions. Voucher specimens of the plants were identified and authenticated at Makerere University Herbarium. Data were analyzed using descriptive statistics, Informant Consensus factor (ICF), Use Reports (URs), paired comparisons, and GraphPad Prism® version 9.0.0 software.

**Results:**

All respondents (*N* = 206, 100%), used plants to induce labour, treat PPH, and induce abortion. One hundred four plant species were documented: most cited or preferred were: *Hoslundia opposita* (*N* = 109, 53%), *Phytolacca dodecandra* (*N* = 72, 35%), and *Commelina erecta* (*N* = 47, 23%). The plants belonged to 49 families, Lamiaceae (16.3%) and Fabaceae (14.3%) having the majority of the species. Herbs were 42 (40%) and trees 23 (22%). Oral administration 95(72%) was the commonest, then topical 19 (14.4%) and vaginal 14(10.6%).

**Conclusion:**

Health surveys revealed that about 27% of deliveries in Uganda take place outside a health facility. Due to the oxytocic effects of plant species reported in this study, they play a triple role of being uterotonics, abortifacients, and treating postpartum haemmorhage. The dilemma lies in the unknown dosages and toxicity levels that could endanger both the mother’s and the unborn child’s lives. Due to Uganda’s high rates of population growth, overall fertility, maternal mortality, and morbidity, policies, and programmes on gendered health provision need to be reevaluated. Integrating herbal medicine into health care systems appears to be a feasible solution.

**Supplementary Information:**

The online version contains supplementary material available at 10.1186/s12905-024-03205-w.

## Introduction

Historically, women have relied on the health benefits of herbal medicine for pregnancies, deliveries, and postpartum care. Like other impoverished countries with sizable populations, Uganda is essentially unable to meet the basic healthcare demands of its female citizens. Thus, maternal mortality and morbidity, represent the most pressing issue [1]. Furthermore, in Uganda, postpartum hemorrhage (PPH) accounts for 25% of all maternal deaths [2], and the majority of deaths due to PPH occur at traditional birth attendants (TBAs). Additionally, unsafe abortion mainly practiced among adolescents and women battling domestic violence also contributes significantly to the high maternal mortality rate. Unsafe abortion in low-income countries accounts for 13% of all maternal deaths [3].

Coupled with the high fertility of Ugandan women, the maternal mortality rate is 336 deaths per 100,000 live births, one of the highest in sub-Saharan Africa [4]. Although most women receive antenatal care (91%), trained healthcare workers [5], supervise only 37% [6]. In rural western Uganda, close to 80% of pregnant women deliver at home [6] with the aid of herbal medicine to induce labour, tone uterus muscles, remove the retained placenta, and manage post-partum bleeding [7].

In addition to the well-established indigenous healthcare system, the widespread use of traditional medicine (TM) in rural Uganda stems from several factors, including sociocultural acceptance of TM, the stigma associated with visiting healthcare facilities because of harsh and rude midwives, the fear of finding out one’s HIV/AIDS status. Despite the pros, there is a wide gap between the continued use of indigenous knowledge of plants in the maternal healthcare and scientific validation of the species being used. Furthermore, not much has been documented in regards to these plant species leading to the loss of this knowledge through cultural erosion and death of custodians. Thus, this study was undertaken to record the indigenous knowledge regarding maternal health to preserve it for the future generation notwithstanding promoting the conservation of the important medicinal plant species. This will act as a lead for the discovery of new drugs.

## Methods

**Fig. 1:**
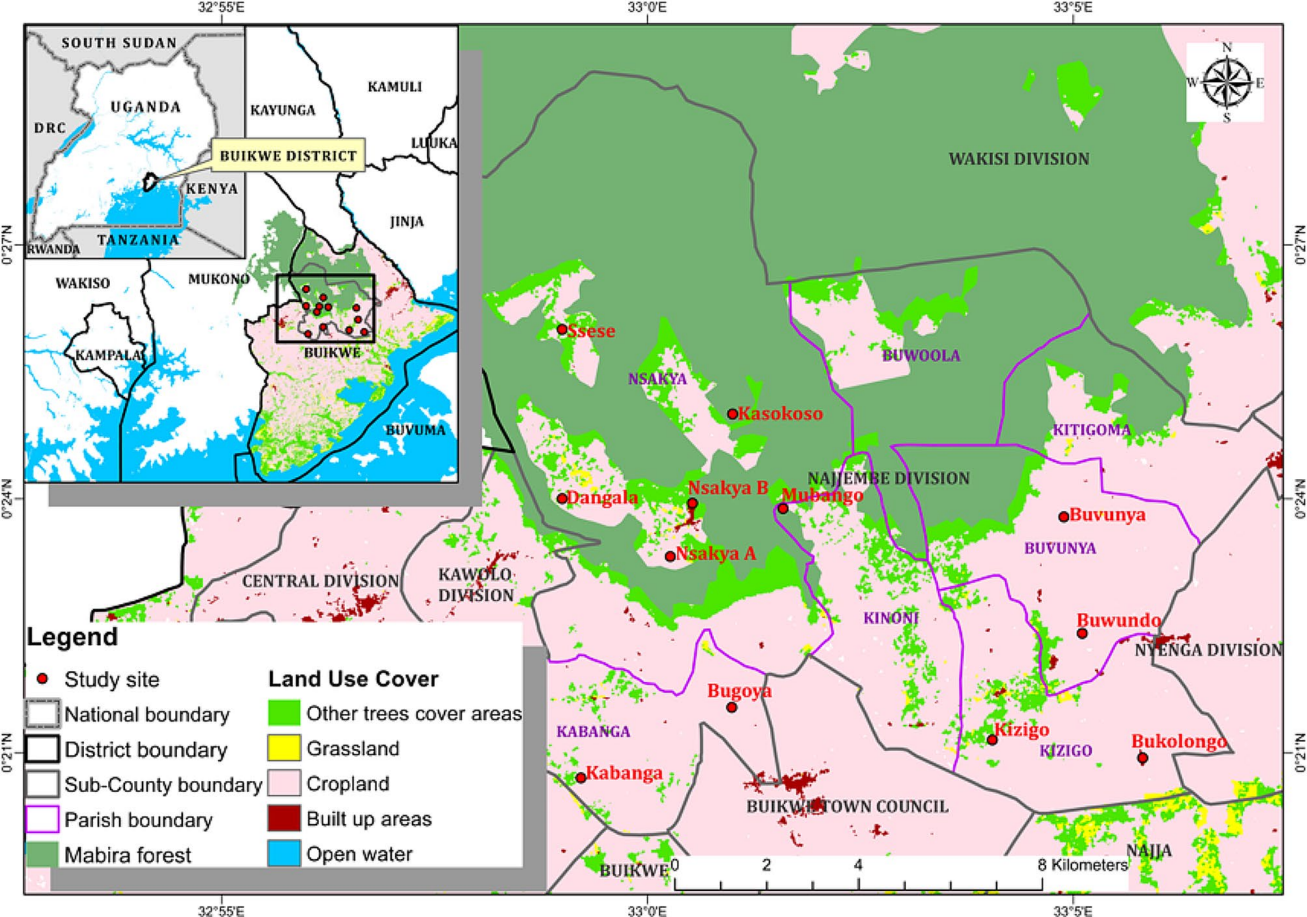
Map of study location showing Najjembe sub-county in Buikwe district

An ethnobotanical survey was carried out in the Najjembe sub-county, Buikwe district (0°20’36.0"N, 33°01’44.0” E), Central Uganda, home to Mabira Central Forest Reserve (Fig. 1) the second largest natural forest in Uganda. Mabira forest is a source of many medicinal plant species and greatly contributes to the livelihoods and survival of the surrounding population. Buikwe District is bordered by Kayunga District to the north, Jinja District to the east, Buvuma District to the southeast, the Republic of Tanzania to the south, and Mukono District to the west. Najjembe sub-county has 8,165 households and houses a population of 33,410 people of which 50.3% are male [4]. In Buikwe district, the vast majority of inhabitants (66.5%) reside in rural areas [4]. Implying more than half of the population are rural dwellers. The district’s economic activities include sugarcane growing and subsistence farming. Eight sub-counties, Nyenga, Najjembe, Ssi Bukunja, Najja, Ngogwe, Buikwe, Kawolo, and Wakisi, as well as two town councils, Njeru and Lugazi, make up the district [8]. Due to the tropical rainforest climate, there are no distinct seasons in the year. In addition to a greater dependence on traditional birth attendants, the Mabira forest reserve’s abundance of medicinal plants has enhanced the women’s indigenous health care system. Consequently, there is a greater dependence on medicinal plants to induce uterine contractions, tonify the uterus, induce abortion, and manage postpartum haemorrhage. Najjembe sub-county served as the study’s location **(**Fig. 1).

### Sample size and sampling procedures

Using the table by Krejcie and Morgan [9] a sample size of 206 respondents was computed based on a population of 440. Four parishes namely; Buvunya, Kabanga, Kizigo, and Nsakya were randomly sampled from which 12 villages (Buvunya, Buwundo, Kabanga, Bugoya, Kizigo, Bukolongo, Ddangala, Kasokoso, Mubango, Nsakya A, Nsakya B, Ssese) were purposively sampled taking into account the adult population. Male and female responders who were at least 18 years old were included in this group.

### Study design, selection of study sites, and participants

All study participants were asked to sign an informed consent form before participation in the study. All methods were carried out following relevant guidelines and regulations as per the School of Health Sciences Ethical Review Board, Makerere University. The field survey was conducted from May – December 2023 using a cross-sectional study design. Seven parishes namely Nsakya, Kabanga, Kinoni, Buwoola, Kitigoma, Kizigo, and Buvunya were randomly selected in Najjembe sub-county and eventually, villages were considered from each parish depending on the population size of the parish, giving a total of 12 villages. **Inclusion criteria of study participants**: Mothers aged 18 and above, Herbalists both men and women, Traditional Birth Attendants these are usually elderly women, then men who always go and collect herbs for their wives and are knowledgeable about traditional medicine (TM). **Exclusion Criteria**: All minors, mothers who do not use plants as medicine for their reproductive health, and women or men who are ignorant about TM. Using snowball sampling, women and men aged 18 and above who had experience in indigenous knowledge of plants used for maternal health were selected. As for key informants, traditional birth attendants and herbalists were identified using the snowballing method based on the principle of saturation [10]. Once a TBA was identified and interviewed, they were asked to refer the research team to another TBA within their networks. The subsequent TBA then referred us to the next TBA in their networks until saturation was reached. The same procedure was followed when choosing herbalists to participate in the study. From each village, 10–15 respondents were interviewed altogether. All respondents first signed consent forms before participating in the study and each respondent was compensated for the time spent participating in the study.

### Ethnobotanical data collection

Ethnobotanical data collection lasted seven months from May – December 2023. Before data collection, a pilot study which lasted one month was undertaken in May 2023 to introduce the study to the local area administration, seek their permission to conduct the study, and pre-test the study tool. Information from the pilot helped in the modification of the final study. Data were collected using a semi-structured questionnaire (Supplementary file 1). All questions that were included in the questionnaire revolved around indigenous knowledge of plants used for maternal health specific to the treatment of postpartum bleeding, inducing uterine contractions, and abortions. Special emphasis was put on the time during and after pregnancy when such botanical remedies were administered. Other nonplant materials that were used for the same cause were also recorded. Six focus group discussions were held with community members to verify the information recorded in the individual interviews. Pairwise ranking was done to get the species that were considered most efficacious by TBAs, mothers, and herbalists. Voucher specimens of the plant species used were collected and taken to the Makerere University Herbarium for identification. Species nomenclature followed the fora for tropical East Africa and was verified using the Plants of the World Online (POWO) database (https://powo.science.kew.org).

### Data analysis

The data were entered into Microsoft Excel, coded, and exported to SPSS software (version 26, SPSS Inc.) for analysis. Descriptive statistics such as percentages and frequencies were used to summarize the data. The informant consensus (ICF) factor was conducted to determine the homogeneity of the medicinal plants’ information collected from the respondents using formula 1 [11]:

$$ ICF=\frac{Nur-Nt}{Nur-1}$$

where “Nur” refers to the total number of use reports (URs) for each condition cluster and “Nt” refers to the total number of species in each use category. The ICF values range from 0 to 1. High ICF values (close to 1) are obtained when only a few plant species are reported to be used by a high proportion of informants to treat a particular disease and this implies that there is a well-defined mechanism in the community of sharing information between informants. When a variety of plant species are utilized to treat the same condition, individuals prefer one over the other. Therefore, preference ranking was performed to determine which species were preferred over others as described in Martin [11, 12]. Key informants were tasked to compare the given botanical remedies based on their values. The most preferred plant species were ranked highest on a scale of 1–5, 5 being the most preferred and 1 being the least preferred.

### Paired comparison of medicinal plants used to manage postpartum bleeding induce uterine contraction and abortion

A paired comparison was made for the five most potent medicinal plants used for the aforementioned conditions in the study area. Following the focus groups, participants were requested to rank the species based on their efficacy in the management of maternal health conditions as follows: 1 = least, 2 = good, 3 = very good, and 4 = excellent [11].

## Results

### Demographic characteristics of the respondents

The socio-demographic characteristics of the study population are shown in Table 1. Two hundred six respondents were interviewed (87.4% female and 12.6% male) on the plants they used to induce uterine contractions, treat postpartum bleeding, and induce abortion. Most of the respondents had attained primary education (51.5%), were Roman Catholic (34.5%), married (70.4%), and 47.4% were business persons. The majority of the respondents were from Nsakya (78.6%) parish and Nsakya A (22.8%), Nsakya B (24.3%), and Mubango (14.6%) villages. Nsakya parish had the highest population thus more villages were sampled as other parishes had very few scattered households.

**Table 1:** Demographic characteristics of the respondents

Characteristic	Frequency	Percentage
Age		
18–24	24	11.7
25–34	75	36.4
35–44	49	23.8
45–54	35	17
55–64	16	7.8
65–74	5	2.4
≥ 75	2	1
Sex		
Female	180	87.4
Male	26	12.6
Education		
No Education	18	8.7
Nursery	1	0.5
Primary	106	51.5
Secondary	79	38.3
Tertiary	2	1.0
Religion		
Anglican	51	24.8
Bishaka - Cult	1	0.5
Islam	50	24.3
Pentecostal	31	15.0
Roman Catholic	71	34.5
Seventh-day Adventist	2	1.0
Marital Status		
Married	145	70.4
Separated	21	10.2
Single	27	13.1
Widowed	13	6.3
Occupation		
Boda-boda rider	4	2.0
Farmer	13	6.6
Businessperson	93	47.4
Herbalist	49	25.0
Administrator	3	1.5
Traditional Birth Attendant	24	12.2
Teacher	8	4.1
Salon attendant	2	1.0

**Fig. 2:**
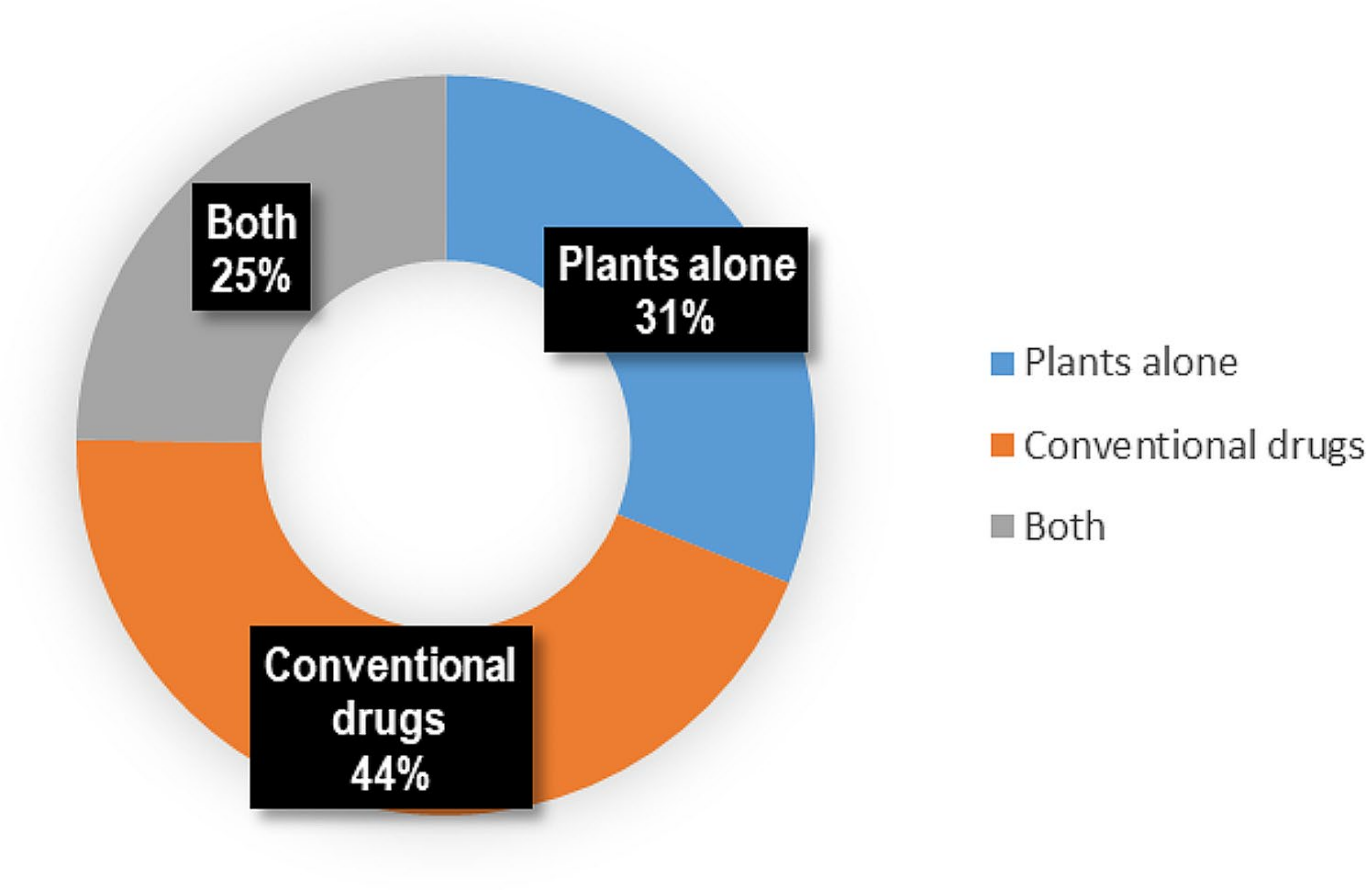
Treatment options used by women in the studied communities of Najjembe sub-county, Buikwe district

Traditional birth attendants reported not to have lost anyone while helping them to give birth. 81% of the respondents reported that they used plants for treating PPH. 83% reported using plants for uterine contractions and 65% reported using plants for abortion. The majority (75%) of the respondents reported using uterotonic plants as a single plant, not in combination. Once the uterus failed to contract, 44.2% of respondents used conventional drugs (Fig. 2).

**Fig. 3:**
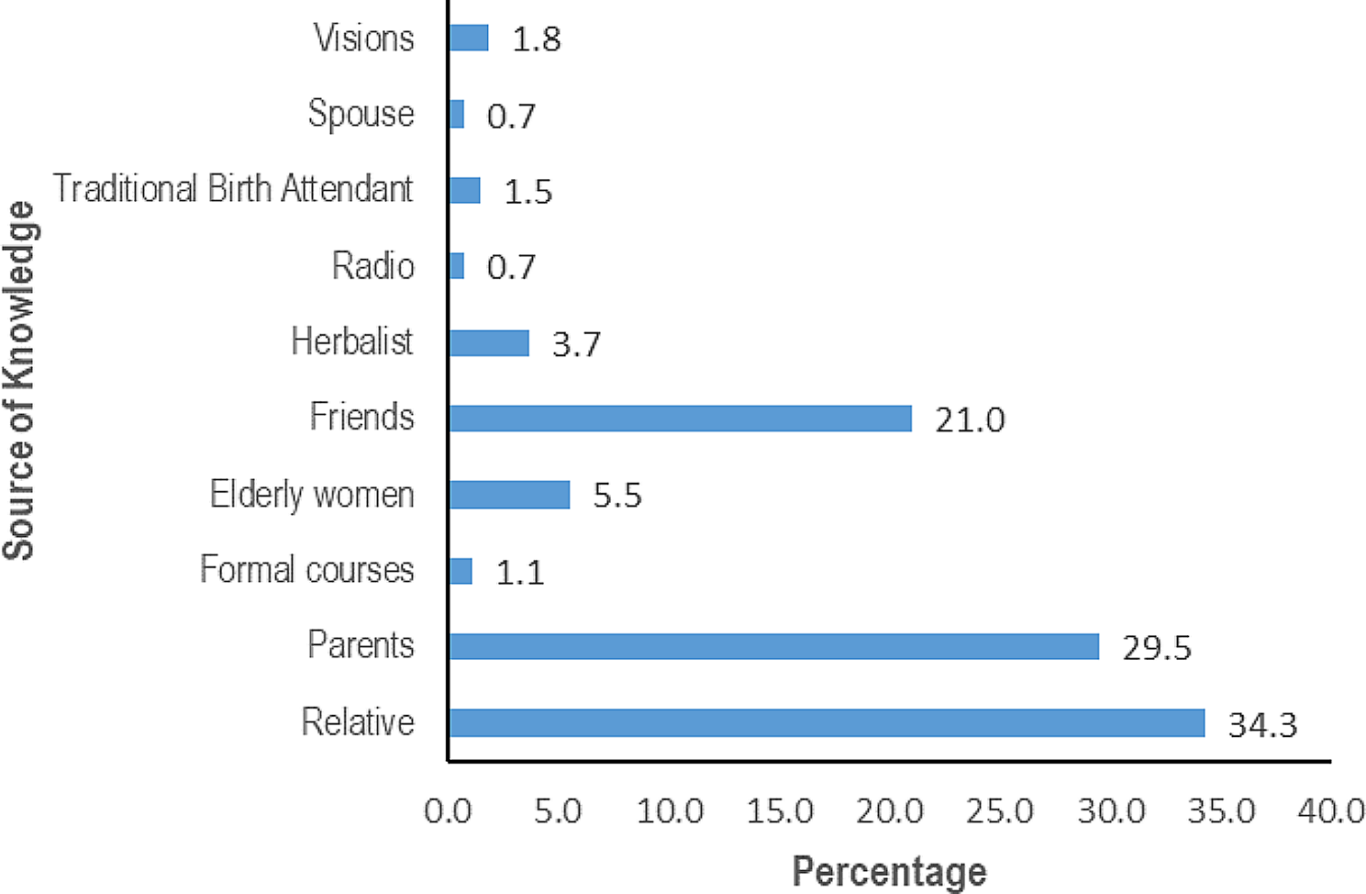
Sources of indigenous knowledge on medicinal plants used by women in Najjembe sub-county, Buikwe district

Relatives (34.3%) were the major sources of knowledge regarding the use of uterotonic plants followed by parents (29.5%) and friends (21%) (Fig. 3). Among the relatives, grandmothers (75.3%) provided the majority of the indigenous knowledge and among the parents, mothers (96.3%) provided the majority of the indigenous knowledge.

**Fig. 4:**
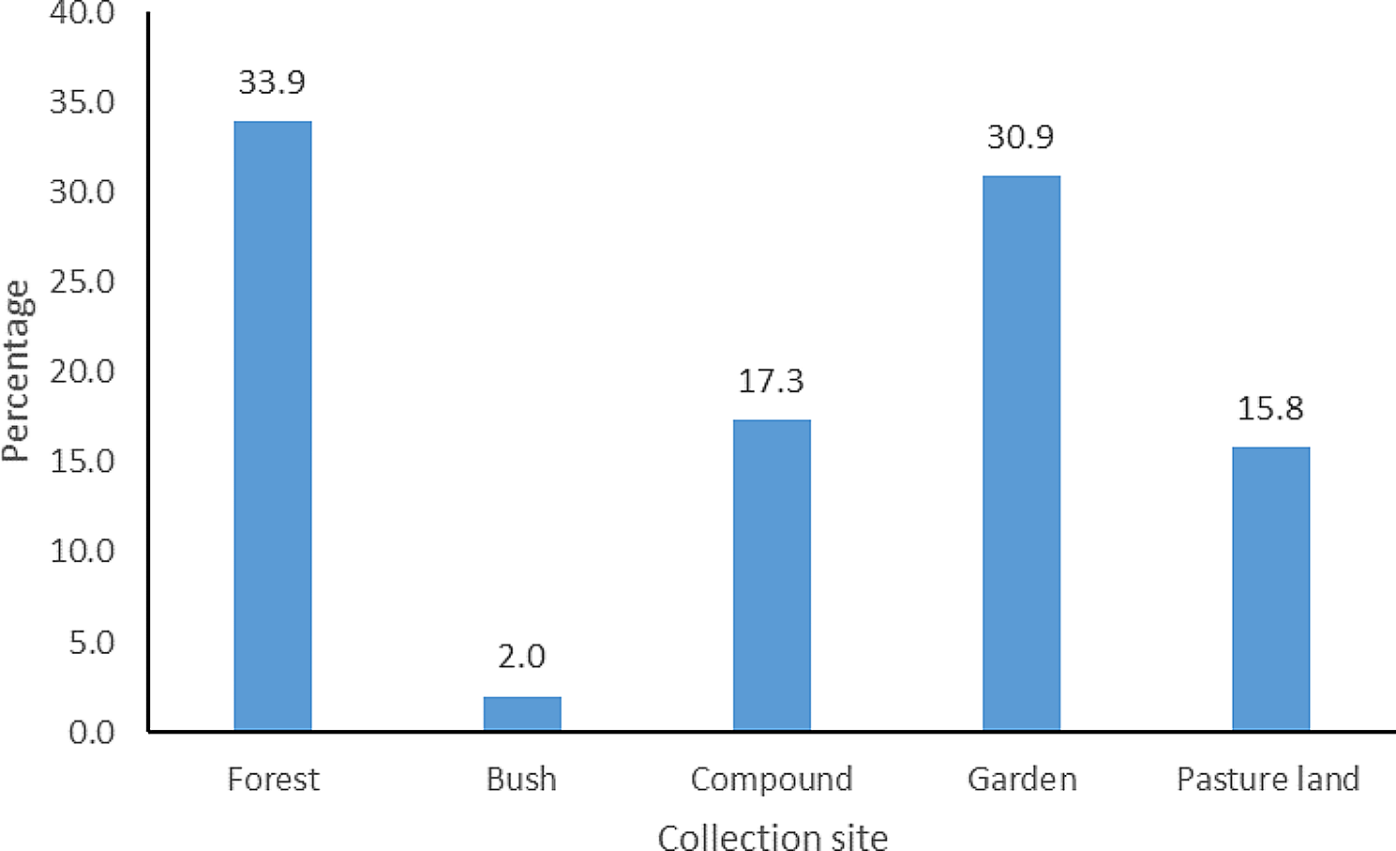
Collection sites for medicinal plants used by women in Najjembe sub-county, Buikwe district

**Fig. 5:**
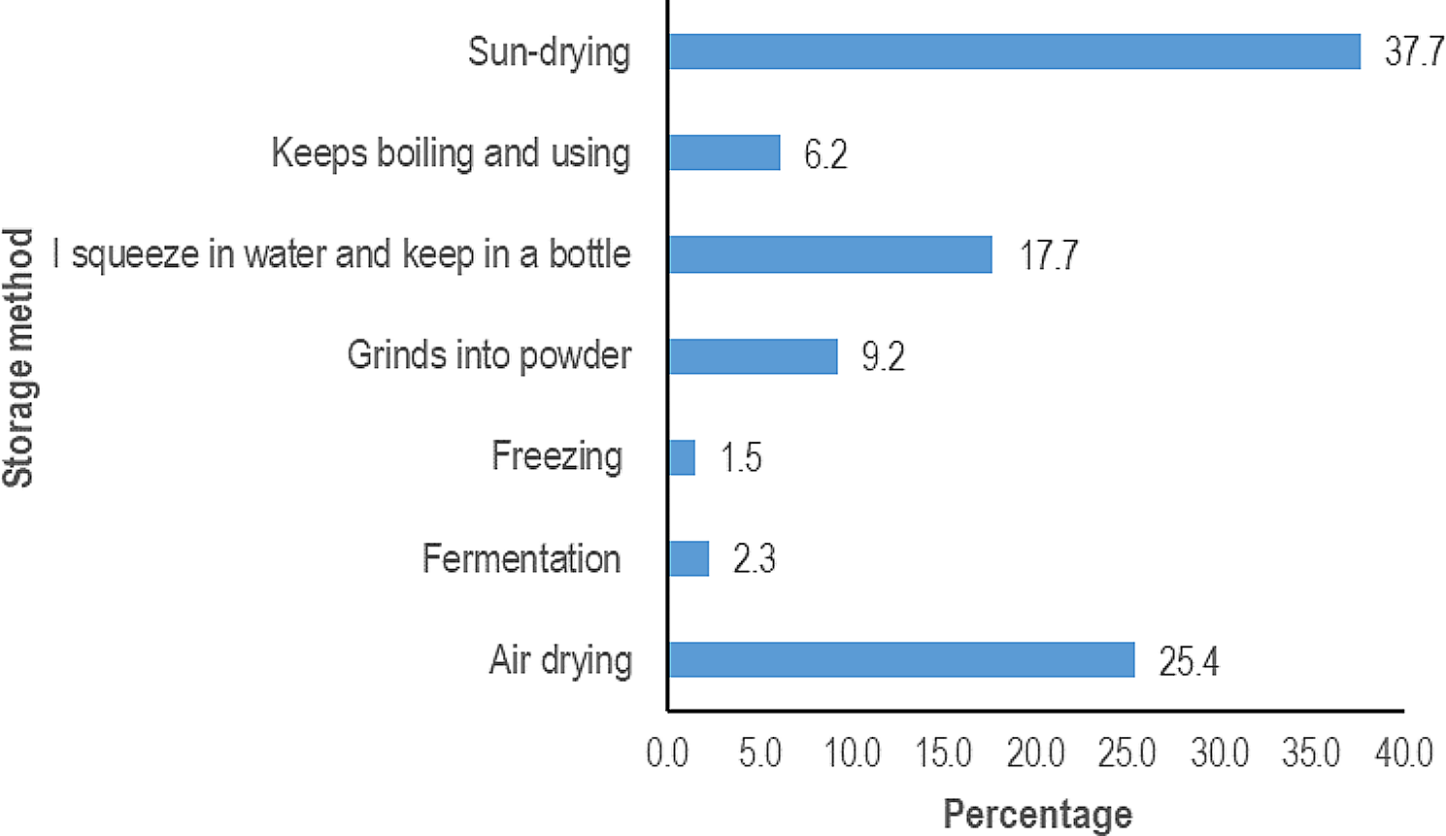
Storage methods for herbal remedies used by women in Najjembe sub-county, Buikwe district

34% of uterotonic plants were collected from the forest followed by 31% from the garden (Fig. 4). The majority of the respondents reported that uterotonic plants were very effective (62.1%) followed by those who said they were effective (37%) and only 1% reported that the plants were not effective at all. The majority of the respondents stored their botanical remedies using traditional storage methods (Fig. 5) but 37% did not store them because they believed they were readily available and could easily be picked from the forest when the need arises.

Whereas some herbal remedies can have tremendous side effects especially uterotonics which can cause uterine rupture and death, 73% of the respondents reported not experiencing any challenges while using uterotonic herbal remedies.

*During focus group discussions, women were asked about the challenges they experienced while using hospital uterotonics. Their responses were;*

*Hospital uterotonics cause dizziness coupled with headaches; they are very expensive; health centers are very far away; inexperienced intern doctors administer uterotonics before delivery time thus causing the child to kill the mother when they kick the heart; they cause hypertension, nausea, pelvic pain, pyrexia, naval and spinal pain, vomiting; sometimes they are very ineffective.*

49% of the respondents grew uterotonic plants. The most commonly grown uterotonic species included; *C. adenocaule*, *P. edulis*, *S. cuneifolia*, *I. batatas*, *P. americana*, *P. prostratus*, *C. papaya*, *C. opulifolium*, *A. esculentus*, *W. ugandensis*, *G. scabra*, *M. esculenta*, *C. gynandra*, *T. riparia*, *C. pepo*, *C. sinensis*, *P. cyaneus*, *L. leonurus*, *A. vera*, *J. exigua*, *C. erecta*, *H. opposita*, *J. curcas*, *Dfragrans*, *V. grantii*, *S. pimpinellifolium*, *S. pimpinellifolium*, *C. frutescens*, *Z. officinale*, *I. spicata*, *N. tabacum*.

Those who did not grow plants reported that they were available in the forest (81.3%) followed by those who said some species would not grow when taken out of the forest (8.1%), those who reported that some species grow on their own were (8.1%) and 2.4% said there is no market for the plants thus no need to grow them. Additionally, 88.8% of the respondents did not buy uterotonic plants from the market. This is majorly because they are available from the wild (forest, bushes, grazing land) (55.2%). Those who went to the market purchased the *A. mauritianum* roots, *C. gynandra* roots, *C. papaya* roots mixed with mud and clay, *A. esculentus* fruits, *W. ugandensis* stembark, *C. adenocaule* leaves, *E. abyssinica* leaves, *P. barbartus* leaves, *H. opposita* leaves, *P. prostratus* leaves.

During focus group discussions, women were asked why they preferred Traditional Birth Attendants to hospitals. The responses were;

*TBAs are polite and give a lot of care to expectant mothers; they provide herbs to induce labour; are nearby and cheap; are very knowledgeable and experienced; provide simple requirements for delivery; give free food; TBAs always do a follow-up and provide post-partum care; they give loans and you pay after delivery; they give enough time to mothers and have emergency deliveries; TBAs help mothers suffering from HIV who are stigmatized from hospitals thus fearing to go there.*

Despite the love and trust for TBAs by mothers, 14.1% reported having lost their relatives and children in the hands of TBAs during delivery.

When asked why mothers did not like going to hospitals for delivery their responses were;

*Hospitals are very expensive and very far; midwives are rude and in most cases beat up mothers, women don’t like taking medication, especially antimalarials e.g. fancida, arm injections e.g. tetanus shots; some women can not afford hospital requirements e.g. disposable plastic bed cover, mama kit (*contains plastic sheeting, razor blades, cotton wool, gauze pad, soap, surgical gloves, exam gloves, cord ties, and a child health card), *hospitals are expensive; fear to test for HIV; intern doctors make mothers push the baby before labor pains which always ends up in killing the mother and baby; poor c-sections resulting in infection of the wound – causing redness, swelling, increasing pain and discharge from the wound, and infection of the womb lining (common); sometimes during C-sections, they cut the uterus, in other cases during C-sections the bladder is injured causing fistula, nurses are so abusive as if you have three or more children already they intimidate you and order you to stop having children, furthermore nurses demand only new hospital requirements e.g. new bedsheets, new baby clothes, new basins which we can not afford.*

**Table 2:** Medicinal plants used for treating postpartum hemorrhage, inducing uterine contractions, and abortion by the women in Najjembe sub-county, Buikwe. District

SN	Family	Species	Plant name	Habit	Condition	Plant part	Preparation method	Administration method	Time	Frequency
1	Papilionaceae	*Indigofera spicata* Forssk., AN105	Mukaliza	Herb	PPH	L	Decoction/ Squeeze in water	Oral	Month 9	10
2	Lamiaceae	*Hoslundia opposita* Vahl, AN106	Kamunye	Shrub	PPH	L	Decoction	Oral	Month 9	109
					Abortion				Month 2, Week 3	1
					UC				Month 7	3
3	Lauraceae	*Persea americana* Mill., AN107	Ovakedo	Tree	PPH	L	Decoction	Oral	Month 9	15
4	Compositae	*Vernonia grantii* Oliv., AN108	Twatwa	Shrub	PPH	L	Decoction	Oral, Topical (Hotpressing belly)	Month 9	6
					Abortion		Decoction	Oral	Month 3, Week 3	1
5	Fabaceae	*Phaseolus vulgaris* L., AN109	Ebijanjalo	Herb	PPH	S	Half cook	Oral	Month 9	1
6	Bromeliaceae	*Ananas comosus* (L.) Merr., AN110	Enanansi ento	Perennial herb	PPH	Fr	Squeeze in water	Oral	Month 9	9
7	Lamiaceae	*Plectranthus cyaneus* Gürke, AN111	Kiwankulata	Herb	PPH	L	Decoction	Oral	Month 9	8
8	Asteraceae	*Melanthera scandens* (Schumach. & Thonn.) Roberty, AN112	Makayi	Herb	PPH	L L	Maceration	Oral	Month 9	2
					Abortion		Squeeze in water		Month 1, Week 4	7
9	Acanthaceae	*Justicia botanica* L., AN113	Nalongo	Herb	PPH	L	Squeeze in water/ Decoction	Oral	Month 9	3
10	Asteraceae	*Senecio discifolius* L., AN114	Mukasa	Herb	PPH	Wh	Squeeze in water	Oral	Month 9	2
					UC	L	Squeeze in water	Vaginal (Sit in)	Month 7	2
					Abortion			Oral	Month 4, Week 1	1
11	Chenopodiaceae	*Chenopodium opulifolium* Koch & Ziz, AN115	Omwetango	Herb	PPH	L	Squeeze in water	Oral/ Topical (Bathing)	Month 9	1
12	Malvaceae	*Sida cuneifolia* Roxb., AN116	Akayeyo akakumirizi	Herb	PPH	R	Squeeze in water	Oral	Month 9	1
					UC		Roast under a charcoal stove and squeeze out fluid	Oral	Month 9	19
13	Lamiaceae	*Leonotis leonurus* (L.) R.Br., AN117	Kifumufumu	Shrub	PPH	L		Oral	Month 9	3
					Abortion		Decoction		Month 4, Week 1	1
14	Caricaceae	*Carica papaya* L., AN118	Paapali	Tree	PPH	R/ L	Roast the Root and squeeze out the fluid/ Decoction for Leaves	Oral	Month 9	1
					UC	R	Raw			3
15	Compositae	*Conyza pyrrhopappa* Sch.Bip. ex A. Rich, AN119	Kafugankande	Herb	PPH	L	Decoction	Oral	Month 9	2
16	Sapindaceae	*Allophylus cobbe* var. dissectus Capuron, AN120	Akatete	Herb	PPH	L	Squeeze in water	Oral	Month 9	1
17	Commelinaceae	*Commelina erecta* Linn., AN121	Enanda	Creeper	PPH	L	Squeeze in water	Oral	Month 9	1
					Abortion	Sap	Raw	Vaginal	Month 1, Week 1	47
18	Rosaceae	*Prunus africana* (Hook) Kalkman, AN122	Entasesa	Tree	PPH	Sb	Decoction	Oral	Month 9	1
19	Asphodelaceae	*Ageratum conyzoides* L., AN123	Namirembe	Herb	PPH	F & L	Decoction	Oral	Month 9	2
					UC	L	Squeeze in water	Topical (Bathing)	Month 9	5
20	Amaranthaceae	*Aerva lanata* (L) Schultes., AN124	Muzuukizi	Herb	PPH	L & F	Decoction	Oral	Month 9	2
21	Asteraceae	*Bidens pilosa* L., AN125	Sere	Herb	PPH	L	Decoction, Squeeze in water	Oral	Month 9	10
22	Sapindaceae	*Cardiospermum halicacabum* L., AN126·	Akambula	Herb	PPH	L	Decoction	Oral	Month 9	1
23	Asphodelaceae	*Aloe vera* (L.) Burm.f., AN127	Ekigajji	Herb	PPH	L	Poultice	Knicker padding	Month 9	1
24	Musaceae	*Musa acuminata* Colla, AN128	Matooke	Perennial herb	PPH	L	Squeeze in water	Oral	Month 9	1
					UC	F	Steaming	Vaginal steaming	Month 8	1
25	Amaranthaceae	*Amaranthus dubius* Thell., AN129	Ddoddo	Herb	PPH	Wh	Decoction	Oral	Month 9	1
					Abortion	R	Crush and add water	Oral	Month 3, Week 1	1
26	Anacardiaceae	*Mangifera indica* L., AN130	Omuyembe	Tree	PPH	Sb	Powder infusion	Oral	Month 9	1
27	Lamiaceae	*Ocimum basilicum* L., AN131	Kakubansiri	Herb	PPH	Sb	Decoction	Oral	Month 9	1
28	Solanaceae	*Capsicum frutescens* L., AN133	Kamulari	Herb	PPH	Fr & L	Maceration	Oral	Month 9	1
					UC	R	Roast the Root and squeeze out the fluid	Oral	Month 9	4
29	Euphorbiaceae	*Jatropha curcas* L., AN134	Ekiloowa	Shrub	PPH	L	Decoction	Oral	Month 9	1
30	Myrtaceae	*Syzygium cumini* (L.) Skeels., AN135	Jambula	Tree	PPH	Sb	Decoction	Oral	Month 9	2
31	Lamiaceae	*Tetradenia riparia* (Hochst.) Codd, AN136	Kyewamala	Shrub	PPH	Leaf sap	Mix with water	Rectal	Month 9	2
					UC	L	Squeeze in water	Vaginal (Sit in)	Month 7	1
32	Piperaceae	*Piper umbellatum* Linn K, AN137	Kigamansole	Herb	PPH	L	Decoction	Oral	Month 9	1
33	Lamiaceae	*Plectranthus prostratus* Gürke, AN138	Mubiri	Herb	PPH	L	Squeeze in water	Oral	Month 9	2
34	Fabaceae	*Erythrina abyssinica* Lam. ex DC., AN139	Jiirikiti	Tree	PPH	Sb	Decoction	Topical	Month 9	1
35	Bignoniaceae	*Kigelia africana* Lam. Benth., AN140	Musa	Tree	PPH	Sb	Decoction	Oral	Month 9	1
36	Polygonaceae	*Oxygonum sinuatum* (Hochst. & Steud. ex Meisn.) Dammer, AN141	Kafumita bagenge	Herb	PPH	L	Squeeze in water	Oral	Month 9	1
37	Euphorbiaceae	*Micrococca mercurialis* (L.) Benth., AN142	Kalyabakyala	Herb	PPH	L	Squeeze in water	Oral	Month 9	1
38	Asteraceae	*Vernonia amygdalina* Del., AN143	Mululuza	Tree	PPH	L	Squeeze in water	Oral	Month 9	1
					UC	L	Squeeze in water	Oral	Month 9	1
39	Vitaceae	*Cyphostemma adenocaule* (A. Rich) Wild. & Drumm, AN144	Akabombo akatono	Climber	PPH	L	Squeeze in water	Oral	Month 9	1
					UC	Wh	Steaming	Vaginal hotpressing	Month 6	14
40	Moraceae	*Artocarpus heterophyllus* Lam., AN145	Ffene	Tree	PPH	L	Decoction	Oral	Month 9	1
41	Asteraceae	*Crassocephalum vitellinum* (Benth.) S.Moore, AN146	Kitonto	Herb	PPH	L & F	Decoction	Oral	Month 9	1
42	Asteraceae	*Sigesbeckia orientalis* L., AN147	Seziwuundu	Tree	PPH	L	Decoction	Oral	Month 9	1
43	Zingiberaceae	*Zingiber officinale* Roscoe, AN148	Ntangawuzi	Herb	UC	Rh	Decoction	Oral	Month 9	4
44	Bignoniaceae	*Markhamia lutea* (Benth.) K.Schum., AN149	Omusambya	Tree	UC	Rb	Decoction	Oral	Month 9	2
45	Crassulaceae	*Kalanchoe pinnata* (Lam.) Pers., AN150	Ekiyondo ekyeru	Herb	UC	L	Powder mix with vaseline	Topical	Month 9	2
46	Passifloraceae	*Passiflora edulis* Sims, AN151	Akatunda akaganda	Climber	UC	R	Raw	Oral	Month 9	1
47	Cleomaceae	*Cleome gynandra* L., AN152	Jobyo	Herb	UC	R	Raw	Oral	Month 9	20
48	Acanthaceae	*Justicia exigua* S. Moore, AN153	Kazunzanjuki	Herb	UC	L	Decoction	Oral	Month 9	3
49	Theaceae	*Camellia sinensis* (L.) Kuntze, AN154	Amajani	Shrub	UC	L	Decoction	Oral	Month 9	2
50 51	Canellaceae Malvaceae	*Warburgia ugandensis* Sprague, AN155 *Abutilon theophrasti* Medik., AN156	Balwejila	Tree	UC	L & Sb	Decoction	Oral	Month 9	2
			Ekifula	Herb	UC	R	Roast the roots and squeeze out the fluid	Oral	Month 9	1
52	Solanaceae	*Nicotiana tabacum* L., AN157	Taaba	Shrub	UC	R	Roast the roots and squeeze out the fluid	Oral	Month 9	1
53	Asparagaceae	*Dracaena fragrans* (L.) Ker Gawl., AN158	Oluwanyi	Shrub	UC	R	Squeeze in water	Oral	Month 9	2
54	Amaranthaceae	*Achyranthes aspera* L., AN159	Mutassuka kkubo	Creeper	UC	L	Squeeze in water	Oral	Month 9	1
55	Musaceae	*Musa paradisiaca* L., AN160	Gonja	Perennial herb	UC	R	Roast the roots and squeeze out the fluid	Oral	Month 9	2
56	Fabaceae	*Abrus precatorius* L., AN161	Olusiiti	Shrub	UC	L	Squeeze in water	Vaginal	Month 8	1
57 58	Convolvulaceae Asteraceae	*Hewittia sublobata* L. Kuntze, AN162 *Taraxacum officinale* (L.) Weber ex F.H.Wigg., AN163	Musota taluma	Climber	UC	L	Raw	Tie around your waist	Month 1	13
			Mavigamukulu	Herb	UC		Decoction	Vaginal steaming	Month 8	1
59	Cucurbitaceae	*Luffa cylindrica* (L.) Rox., AN164	Kyangwe	Climber	UC	Fr	Burn to charcoal	Topical (Rub from your waist down to the thighs)	Month 7	1
60	Asphodelaceae	*Aloe vera* (L.) Burm.f., AN165	Ekigaji	Sedge	UC	L	Powder mix with vaseline	Topical (Rub from your waist down to the thighs)	Month 7	12
61	Basellaceae	*Basella alba* L., AN166	Nderema	Climber	UC	L	Squeeze in water	Oral	Month 7	3
62	Convolvulaceae	*Ipomoea batatas* (L.) Lam., AN167	Lumonde	Creeper	UC	L	Squeeze in water	Vaginal (Sit in)	Month 7	25
63	Lamiaceae	*Plectranthus prostratus* Gürke, AN168	Mubiri	Creeper	UC	Wh	Squeeze in water	Oral	Month 7	8
64	Cucurbitaceae	*Mormodica feotida* Schumach, AN169	Bombo	Climber	UC	F	Powder infusion	Oral	Month 7	7
					UC	S & Fr	Powder mix with vaseline	Topical (Rub from your waist down to the thighs)	Month 7	
65	Musaceae	*Musa acuminate* Cavendish Subgroup, AN170	Bogoya	Perennial herb	UC	P	Raw	Oral	Month 8	2
66	Menispermaceae	*Cissampelos pareira* L., AN171	Akavawala	Herb	UC	L	Tie it around the waist	Topical	Month 6	1
67	Poaceae	*Sorghum bicolor* (L.) Moench, AN172	Omuwemba	Grass	UC	G	Smoking	Vaginal	Month 8	1
68	Fabaceae	*Crotalaria spinosa* Hochst., AN173	Kasambandegge	Herb	UC	L	Powder mix with vaseline	Topical (Rub from your waist down to the thighs)	Month 8	11
69	Burseraceae	*Canarium schweinfurthii* Engl., AN174	Omuwaffu	Tree	UC	Sb	Powder mix with vaseline	Topical (Rub from your waist down to the thighs)	Month 8	4
70	Apocynaceae	*Rauvolfia vomitoria* Afzel., AN175	Kamwanyimwanyi akokutale	Herb	UC	L	Powder mix with vaseline	Topical (Rub from your waist down to the thighs)	Month 9	1
71	Malvaceae	*Abelmoschus esculentus* (L.) Moench, AN176	Bamia	Herb	UC	Fr	Decoction	Oral	Month 8	10
72	Asparagaceae	*Dracaena steudneri* Engl., AN177	Kajjo lyanjovu	Tree	UC	Sb	Decoction	Oral/ Vaginal (Sit in)	Month 8	1
73	Sapindaceae	*Cardiospermum halicacabum* L., AN178	Akambula	Herb	UC	L	Squeeze in water	Topical (Bathing)	Month 7	7
74	Lamiaceae	*Tetradenia riparia* (Hochst.) Codd, AN179	Omulavumba	Herb	UC	L	Powder mix with vaseline	Topical	Month 8	1
75	Cucurbitaceae	*Cucurbita pepo* L., AN180	Nsujju	Creeper	UC	R	Raw	Oral	Month 7	1
					Abortion		Decoction	Oral	Month 1, Week 1	1
76	Mimosaceae	*Newtonia buchananii* (Baker) Gilb. & Perr., AN181	Empewere	Herb	UC	L	Decoction	Oral	Month 8	1
77	Rosaceae	*Prunus africana* (Hook.f.) Kalkman, AN182	Entasesa	Tree	UC	L	Decoction	Oral	Month 8	1
78	Poaceae	*Saccharum officinarum* L., AN183	Kikajjo	Grass	UC	P	Smoking	Vaginal smoking	Month 8	4
79	Apiaceae	*Centella asiatica* (L.) Urban, AN184	Kabo kabakyala	Creeper	UC	L	I squeeze in water	Topical (Bathing)	Month 7	1
80	Fabaceae	*Erythrina abyssinica* Lam. ex DC., AN185	Jirikiti	Tree	UC	L	Smoking	Vaginal smoking	Month 8	3
81	Moraceae	*Ficus natalensis* Hochst., AN186	Mutuba	Tree	UC	R	Decoction	Oral	Month 7	4
82	Talinaceae	*Talinum portulacifolium* (Forssk.) Asch. ex Schweinf., AN187	Empoza	Herb	UC	L	Powder mix with vaseline	Oral/ Topical (Rub your waist down to the thighs)	Month 7	1
83	Euphorbiaceae	*Jatropha curcas* L., AN188	Ekirowa	Climber	UC	L	Squeeze in water	Topical (Bathing)	Month 9	1
84	Moraceae	Ficus exasperata Vahl, AN189	Oluwawu	Tree	UC	L	Powder infusion	Oral	Month 7	1
85 86	Commelinaceae Asteraceae	*Commelina erecta* Linn., AN190 *Guizotia scabra* (Vis.) Chiov., AN191	Ennanda	Creeper	UC	Sap	Raw	Cervical piercing	Month 9	2
			Kilalankuba	Herb	UC	L	Decoction	Oral	Month 9	1
87	Amaranthaceae	*Amaranthus dubius* Mart. ex Thell., AN192	Ebooge	Herb	UC	L	Squeeze in water	Vaginal (Sit in)	Month 8	1
88	Phytolaccaceae	*Phytolacca dodecandra* L’Hér., AN193	Oluwoko	Shrub	Abortion	L, R	Crush and add water	Oral	Month 1, Week 4	72
89	Theaceae	*Camellia sinensis* (L.) Kuntze, AN194	Amajani	Shrub	Abortion	L	Decoction	Oral	Month 1, Week 4	7
90	Canellaceae	*Warburgia ugandensis* Sprague, AN195	Balwejira	Tree	Abortion	Sb	Decoction	Oral	Month 1, Week 4	13
91	Acanthaceae	*Justicia botanica* L., AN196	Nalongo	Shrub	Abortion	L	Decoction	Oral	Month 1, Week 4	1
92	Euphorbiaceae	*Manihot esculenta* Crantz, AN197	Muwogo	Shrub	Abortion	L	Decoction	Oral	Month 3, Week 1	1
93	Asparagaceae	*Dracaena fragrans* (L.) Ker Gawl., AN198	Omulamula	Shrub	Abortion	R/ St	Crush and add water	Oral	Month 4, Week 1	1
94	Solanaceae	*Solanum incanum* L., AN199	Entengotengo	Shrub	Abortion	R/ St	Crush and add water	Oral	Month 2, Week 4	1
95	Rosaceae	*Prunus africana* (Hook) Kalkman, AN200	Entasesa	Tree	Abortion	Sb	Decoction	Oral	Month 4, Week 1	3
96	Fabaceae	*Senna occidentalis* (L.) Link, AN201	Mutanjoka	Grass	Abortion	R/ St	Decoction	Oral	Month 4, Week 1	2
97	Rutaceae	*Citrus sinensis* (L.) Osbeck, AN202	Omucuungwa	Tree	Abortion	R	Crush and add water	Oral	Month 3, Week 4	1
98	Poaceae	*Axonopus aureus* P.Beauv., AN203	Pasikalamu	Shrub	Abortion	L	Squeeze in water	Oral	Month 1, Week 1	1
99	Fabaceae	*Erythrina abyssinica* Lam. ex DC., AN204	Ejirikiti	Tree	Abortion	Sb	Decoction	Oral	Month 2, Week 3	1
100	Solanaceae	*Solanum pimpinellifolium* L., AN205	Obunyanya obuganda	Herb	Abortion	L	Squeeze in water	Oral	Month 2, Week 1	1
101	Euphorbiaceae	*Euphorbia heterophylla* Linn. Klotzsch & Garcke, AN206	Kisandasanda	Herb	Abortion	L	Decoction	Oral	Month 4, Week 1	1
102	Apocynaceae	*Alstonia boonei* De Wild., AN207	Mubajjangalabi	Tree	Abortion	Sb	Decoction	Oral	Month 4, Week 1	1
103	Menispermaceae	*Cissampelos mucronata* A. Rich., AN208	Kavamaggombe	Herb	Abortion	R	Crush and add water	Oral	Month 4, Week 4	1
104	Solanaceae	*Solanum incanum* L., AN209	Akatengotengo akatono	Shrub	Abortion	R	Crush and add water	Oral	Month 2, Week 1	1

**Key**: Plant parts; Flowers – F, Leaves – L, Stem – St, Roots – R, Fruits – Fr, Stembark – Sb, Rb – Rootbark, Wh – Whole, P – Pseudostem; Condition: PPH – Postpartum hemorrhage, UC – Uterine Contraction

**Table 3:** None plant materials used for treating postpartum hemorrhage and inducing uterine contractions

S/*N*	Material	Condition	Preparation method	Administration method	Time	Frequency
1.	Snail shell	Uterine contraction	Roast and grind to powder, mix with vaseline	Topical (Rub from pelvic region down to the thighs)	Month 7	15
2.	Egg Shell		Roast and grind to powder, mix with vaseline	Topical (Rub from pelvic region down to the thighs)	Month 7	2
3.	Cow dung		Smoking	Vaginal smoking	Month 7	5
4.	Snakeskin		Dry and grind to powder, mix with vaseline	Topical (Rub from your waist down to the thighs)	Month 8	3
5.	*Medusomyces gisevii* L.	Postpartum hemorrhage	Fermentation	Oral	Month 9	1

**Fig. 6:**
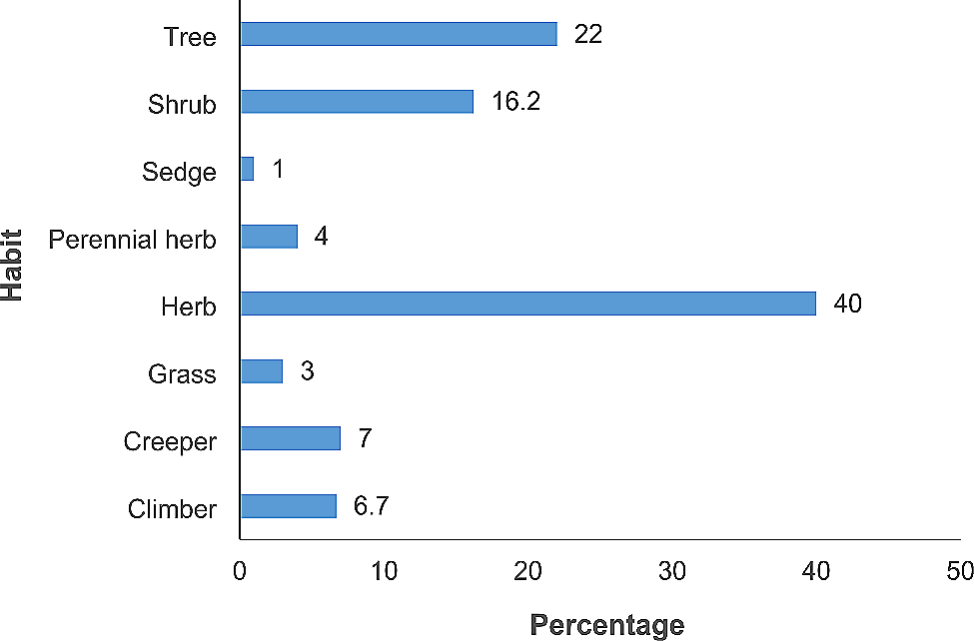
Habits of plants used by the women in Najjembe subcounty, Buikwe district

**Fig. 7:**
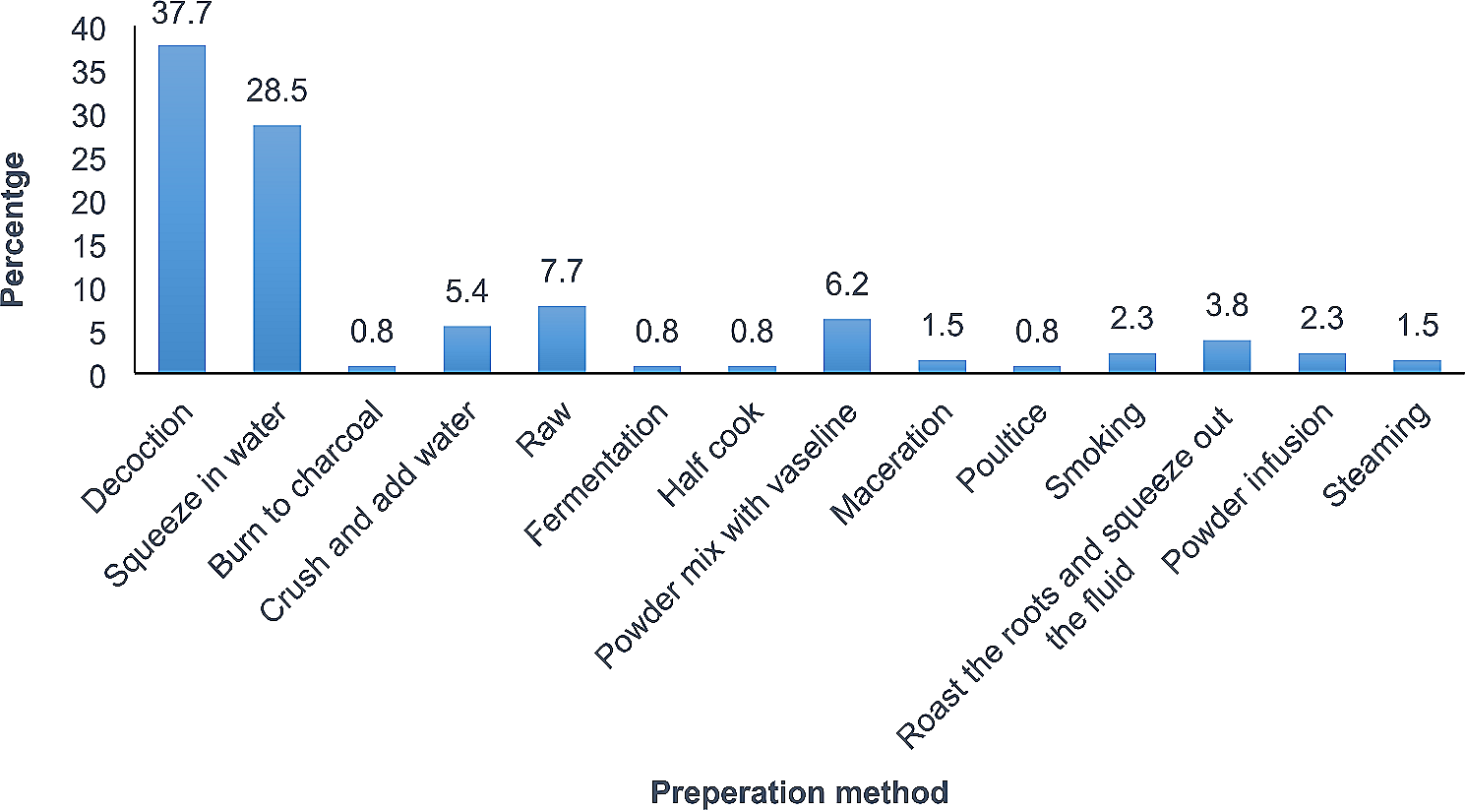
Preparation methods for botanical remedies used by women in Najjembe sub-county, Buikwe district

**Fig. 8:**
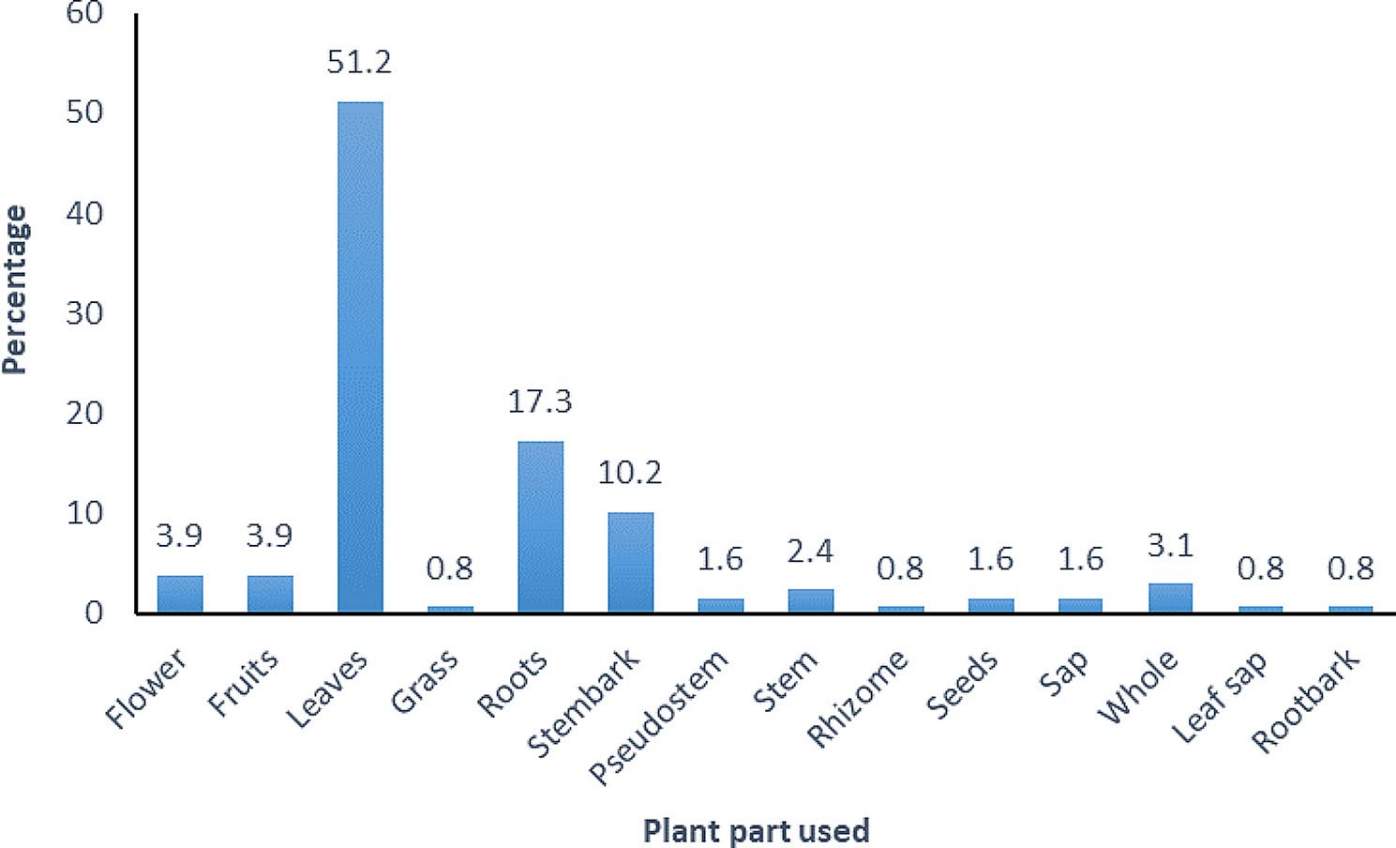
Plant parts used in the preparation of botanical remedies by women in Najjembe sub-county, Buikwe district

**Fig. 9:**
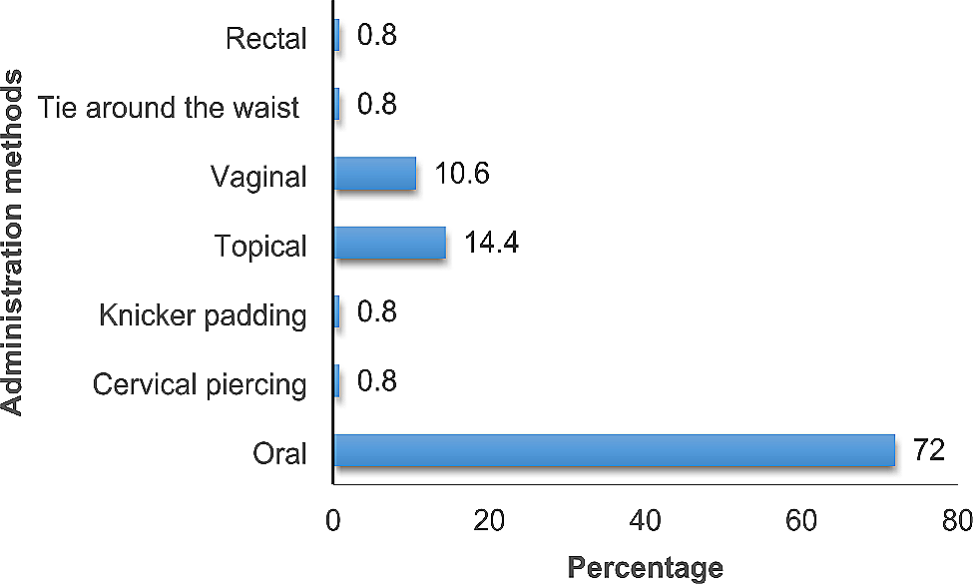
Methods of administration for botanical remedies used by women in Najjembe sub-county, Buikwe district

The species used for postpartum hemorrhage, uterine contraction, and abortion belonged to 49 families and 104 genera (Table 2). The majority of the species belonged to the family Lamiaceae (16.3%) followed by Fabaceae (14.3%). *Hoslundia opposita* was the most frequently used species with 109 mentions. The high frequency arises from its use for treating postpartum hemorrhage, cleansing the uterus after birth, healing vaginal tears and stiches after birth, and is thus considered extremely effective by most mothers. In addition to the diversity of plant species used in maternal care by the women in Najjembe sub-county, none plant materials (snail shells, egg shells, cow dung, and snakeskin) for inducing uterine contractions, and *Medusomyces gisevii* L. mushroom was also used for treating postpartum hemorrhage (Table 3). According to preference ranking, *Aloe vera* was most preferred followed by *Capsicum frutescens* (Table 4). From the pairwise ranking of the five most effective plants, *Hoslundia opposita* was the best with a score of 42. (Table 5). Most species used in the herbal preparations grew as herbs (40%), followed by trees (21.9%) and shrubs (16.2%). (Fig. 6). The majority of the herbal preparations were made using leaves (51.2%) followed by roots (17.3%) and stembark (10.2%) (Fig. 7). Majority of the herbal preparations were prepared by decoction (37.7%) followed by those they squeezed in water (28.5%) (Fig. 8). They were then administered orally (72%) followed by topically (14.4%) and through the vagina (10.6%) (Fig. 9).

**Table 4:** Preference ranking of medicinal plants used by the women in Najjembe sub-county, Buikwe district

	Respondents							Score	Rank
	*R*_1_	*R*_2_	*R*_3_	*R*_4_	*R*_5_	*R*_6_	*R*_7_		
*Commelina erecta*	3	5	3	5	3	3	4	24	3rd
*Hoslundia opposita*	5	4	4	4	5	4	4	30	1st
*Ipomoea batatas*	3	5	2	3	3	3	3	21	4th
*Cleome gynandra*	3	4	3	2	2	3	3	20	5th
*Phytolacca dodecandra*	4	3	3	4	4	4	5	27	2nd
*Sida cuneifolia*	3	2	2	3	4	3	2	19	6th

**Table 5:** Paired comparison of five most commonly used medicinal plants used by women in Najjembe sub-county, Buikwe district

	Respondents										Score	Rank
	*R*_1_	*R*_2_	*R*_3_	*R*_4_	*R*_5_	*R*_6_	*R*_7_	*R*_8_	*R*_9_	*R*_10_		
*Commelina erecta*	3	4	3	3	2	3	4	2	3	4	31	3rd
*Ipomea batatas*	3	2	3	2	3	3	2	2	2	3	25	4th
*Hoslundia opposita*	4	5	4	4	4	4	4	5	4	4	42	1st
*Cleome gynandra*	2	3	2	2	2	3	2	2	2	2	22	5th
*Phytolacca dodecandra*	4	3	3	4	4	4	3	3	4	4	36	2nd

Despite the significant contribution of the plant species in Table 2 to women’s reproductive healthcare, a number of side effects (Table 6) were reported by the mothers on excess consumption of these herbs or on the consumption of the herbs before labour contractions commenced.

**Table 6:** Side effects caused by some of the plant species used for treating postpartum hemorrhage, inducing uterine contractions, and abortion by the women in Najjembe sub-county, Buikwe

SN	Species	Side effects
1	*Zingiber officinale* Roscoe	Elevates blood pressure
2	*Rauvolfia vomitoria* Afzel.	Elevates blood pressure
3	*Phytolacca dodecandra* L’Hér.	Excessive bleeding
		Death
		Ejecting/ removing the uterus
4	*Commelina erecta* Linn.	Wounds in the uterus
		Rotting of the cervix
		Over bleeding following its use for abortion
		Death
5	*Cleome gynandra* L.	If taken before contractions begin, the baby gets tired and thus could die during pushing
		Leaking of the amniotic fluid
6	*Melanthera scandens* (Schumach. & Thonn.) Roberty	Excessive bleeding
7	*Warburgia ugandensis* Sprague	Ejecting/ removing the uterus
8	*Camellia sinensis* (L.) Kuntze	Uterine rupture
9	*Abutilon theophrasti* Medik.	Uterine rupture
		Death
10	*Carica papaya* L.	The uterus can fail to open leading to death
11	*Musa paradisiaca* L.	Once used and fails to work it leads to death
13	*Ficus natalensis* Hochst.	Elevates blood pressure
14	Nicotiana tabacum L.	Too much before labour pains, you get wounds in the uterus
15	*Indigofera arrecta* Hochst. ex A.Rich.	Too much leads to leaking of the amniotic fluid
16	*Markhamia lutea* (Benth.) K.Schum.	Too much can lead to loss of blood while giving birth
17	*Prunus africana* (Hook.f.) Kalkman.	Death
18	*Cardiospermum halicacabum* L.	Excessive bleeding

## Discussion

The process of giving birth brings joy to the family but prenatal and postnatal care is key to the survival and health of the mother and baby. Thus, this study highlights the significant contribution of indigenous plant species to the reproductive health of women in marginalized communities and more so in low-income countries. It goes further to point out the deleterious side effects of some of these species upon consumption in excess amounts or on consumption before labour pains commence.

Additionally, non-plant materials (egg shells, cow dung, snakeskin, and mushroom - *Medusomyces gisevii* ) were also reported to have oxytocic properties. Medicinal plants used for labour induction were used from seven months until the end of the gestation period (normally 9 months) or at the onset of labour pains. Plants that induce uterine contractions have similar action as that of oxytocin hormone, produced on the posterior lobule of the hypophysis, which stimulates the uterus and causes strong contractions, thus inducing labour [13]. Since uterine atony is the major cause of postpartum bleeding [14], plants that induce labour in addition to inducing or augmenting labor are also used in the treatment of post-partum hemorrhage [15]. Post-partum hemorrhage is the leading single direct cause of maternal mortality worldwide [16]. Thus the doubled role played by these plant species emphasizes the importance of documenting and conserving them.

Abortion is considered a sin according to religious teachings but is also considered an abomination in the majority of African cultures. Nonetheless, legal abortions are accepted on the grounds that there is a risk to the life or health of the pregnant woman if the pregnancy results from rape, incest or sexual violence, severe or fatal fetal anomaly, or socio-economic grounds. However, these grounds can be expressed in legal texts in ‘vague and confusing’ ways, making them even more difficult to implement [17]. Furthermore, several United Nations human rights bodies have recognized the deleterious impact of restrictive abortion laws on women’s health [18–20] and have consistently raised general concerns about the inaccessibility of safe abortion services. Results of this study showed that the majority of the women’s reasons for abortion were domestic violence and neglect from the spouses-to-be. This shows a gap in the social support especially councilling services at the village level. The respondents also noted that some uterotonic plants could serve as abortifacients for the case of *Hoslundia opposita*, *Senecio discifolius*, and *Cucurbita pepo*.

The famous “*Emumbwa”* is another traditional medicine you can not miss to find in every pregnant woman’s home. *“Emumbwa”* is a mixture of different herbs and clay which form a bar when dry. The clay in “*emumbwa*” is believed to contain minerals like iron and calcium, and this bar preserves the herbs mixed into it and thus can be stored for over a year without going bad. Many pregnant women in Uganda drink this clay and herb concoction to manage morning sickness, treat illnesses including malaria, syphilis, candida, and induce uterine contractions. Some believe the herbs can alter the sex of a child and cleanse them from curses. Women get “*emumbwa*” from TBAs though the majority buy them from herbalists, and herbal medicine vendors, who live in towns and cities [7].

Uterotonic plants have stood the test of time among locals but the childbirth process can not rely on the use of plants alone as women may have several diseases and complications that require modern hospitals. Additionally, some women may not know the correct month to start taking oxytocic plants thus they can end up taking them during the early months of pregnancy thereby inducing an abortion [13]. Whereas TBAs are highly trusted by the village women, the majority of the TBAs are very old women living in small dirty huts and with poor eyesight. This puts at risk the survival of the mother and the unborn child. On that note, women noted the major cause of death during childbirth in Najjembe is PPH due to uterine atony and this usually occurs at TBAs. Giving birth at TBAs and at home increases the vulnerability of poor, rural women to post-partum hemorrhage [21]. Some plant species documented in this study were found to be used elsewhere in Uganda and other countries. For example, the women in Najjembe chewed the raw roots of *Cleome gynandra* to induce labour. According to Oryem et al. [22], *Cleome gynandra* is widely used in hastening childbirth. A herbal drug made up of *Cleome gynandra* is used widely to fasten childbirth [23]. Cleome gynandra roots are chewed in Western Uganda to induce labour [6].

The methanolic extract of *Bidens pilosa* has been reported to show weak uterine stimulant effects on the guinea pig uterus in Rwanda [24]. Women in western Uganda smoke *B. pilosa* in a pipe or drink the water extract to induce labour [6]. *Bidens pilosa* aqueous and methanolic extracts increase uterine motility and strongly augment oxytocin activity although it has a weak uterine stimulating activity [25]. *Vernonia amygdalina* may indeed contain a potent uterotonic agent, since aqueous extracts (100 mg/mL) induced uterine contraction amplitudes in guinea pig dams that were similar to those of ergometrine [26]. Another study showed that the extracts of *Commelina africana*, *Sida corymbosa* and *Vernonia amygdalina* yielded the biggest increases in contractility in the uterine model, i.e., 31.8% at 210 min, 32.8% at 210 min, and 28.3% at 150 min, respectively [27] and maintained the contractile effect for 2.5–3.5 h, suggesting an added benefit in terms of being long-acting and having a sustained uterotonic action. *Vernonia amygdalina* roots are chewed to induce labour [6]. *Vernonia amygdalina* and *Ocimum gratissimum* are regularly consumed as soup during pregnancy to promote easy progression, strengthen or tone the uterus muscle, and prevent complications, such as pain, bleeding, and abortion [28]. However, during labor, these two plants are administered fresh as squeezed leaves or aqueous extracts same as the women in this study.

*Carica papaya* latex induces spasmodic (tetanic spasms) contraction of the uterus muscle, similar to oxytocin and prostaglandin F2α [29], and the roots are chewed to induce labour [6] which is in agreement with this study. *Commelina erecta* is inserted in the vagina to induce labour [6]. The majority of the women in Najjemebe reported that the sap was very potent for abortion as it opened up the cervix within one hour. Nonetheless, due to the high potency, many women die in the process due to over-bleeding and strong pains. *Luffa cylindrica* aqueous leaf extract ruptures uterine membranes thereby causing strong (oxytocic) uterine contractions that are immediate and stable for more than 30 min thereby speeding up labour during childbirth [30]. Whereas some plant species have been reported by other authors as inducers of uterine contractions, in this study they played a double role in inducing labour and treating postpartum hemorrhage. Diterpenes, phenylpropanoid glucosides, heterocyclic aldehydes, fatty acids, saponins, sterols, and polypeptides have been reported to be responsible for the uterotonic activity of the oxytocic plants [27].

The high maternal mortality rate in Uganda and other low-income countries means many of these women did not receive the necessary maternal care due to socio-economic factors [21], but they have access to medicinal plants, which potentially could save them [31]. Therefore, documentation and biological identification of traditionally used herbal remedies is an ideal starting point for biological target-oriented drug discovery efforts and their pharmacological characterization may eventually lead to the development of novel uterotonic drugs thereby improving the reproductive health of women with low access to primary healthcare [27].

## Conclusion

This study presents a high diversity of plant species embedded in the indigenous healthcare system of women living in Najjembe sub-county, Buikwe district, Uganda. The continuous and trusted use of plants by women, herbalists, and traditional birth attendants in inducing labour, abortion, and treating postpartum hemorrhage is an indicator that these herbs are potent and could guide the discovery of novel oxytocics. Additionally, the deleterious side effects caused by overconsumption and consumption of these species before labour pains commence have also been reported. Thus, a need for field and laboratory research to establish the appropriate dosage, toxicity, and efficacy levels to address the current crisis and mysteries surrounding maternal and infant mortality in Uganda [6]. Abortifacient plants with their harmful effects have not yet been studied thoroughly thus the a need for a thorough examination of their plant extracts to test them for potential toxicity and mutagenicity [15]. Scientific investigations on plants that contribute to maternal health of marginalised populations can guide the formation of informed health policies, guide safe motherhood programmes, as well as collaborative approaches involving training traditional birth attendants thereby equipping them further. This study also noted the need to set up counseling services for adolescents and mothers as the most noted causes of abortion were failed relations and domestic violence.

### Electronic supplementary material

Below is the link to the electronic supplementary material.

Supplementary Material 1

## Data Availability

All data is provided within the manuscript or supplementary information files attached.

## Abbreviations

F: Flowers
Fr: Fruits
ICF: Informant consensus factor
L: Leaves
P: Pseudostem
PPH: Postpartum hemorrhage
R: Roots
Rb: Rootbark
St: Stem
TBAs: Traditional birth attendants
UC: Uterine contraction
URs: Use Reports
Wh: Whole
