# Supplementary material for: Understanding maternal Ethnomedical Folklore in Central Uganda: a cross-sectional study of herbal remedies for managing Postpartum hemorrhage, inducing uterine contractions and abortion in Najjembe sub-county, Buikwe district
Source: BMC Womens Health. 2024 Jun 17;24:349. doi: 10.1186/s12905-024-03205-w (PMC11181598; doi:10.1186/s12905-024-03205-w)
Supplement: Supplementary file 1 — Supplementary Material 1 [file 12905_2024_3205_MOESM1_ESM.docx]

**UTEROTONIC AND POSTPARTUM HAEMORRHAGE PLANTS USED BY THE WOMEN IN NAJJEMBE SUB COUNTY, BUIKWE DISTRICT**

**Introduction**

My name is.................from Makerere University. We are researching uterotonic and postpartum haemorrhage plants. In Uganda, many women die from postpartum haemorrhage and young girls die too from abortions. This has resulted in an increased maternal mortality rate. I am interested in learning about the plants used to (i) stop postpartum haemorrhage, (ii) induce uterine contractions, (ii) cause abortion, and (iii) used for postpartum care.

I would like you to participate in this interview. If you choose to take part, I will not use your real name, and what you tell me will only be used for my research.

This interview will take around 40 minutes – would you like to take part? Yes [ ] No [ ]

**PART 1: Socio-Economic Characteristics of the Respondents**

**District:** Buikwe **Sub-county:** Najjembe

**Parish:** ………………. **Village:** …………………

***Capture GPS location (Mandatory)***

1. When were you born? (MM/DD/YY) .........................................
2. **Sex:** Male [ ] Female [ ]
3. **Education:** No Education [ ] Primary [ ] Secondary O ‘Level [ ] Secondary A’Level [ ] Tertiary [ ]
4. **Religion:** Roman Catholic [ ] Anglican [ ] Seventh-day Adventist [ ] Pentecostal [ ] Islam [ ] Atheism [ ] Others specify……….
5. **Marital status:** Married [ ] Single [ ] Divorced [ ] Separated [ ] Widowed [ ] Others specify…….

6a. **Occupation:** Businessperson [ ] Farmer [ ] Boda-boda rider [ ] Teacher [ ] Nurse [ ] Salon attendant [ ] Herbalist [ ] Traditional Birth Attendant [ ] Administrator e.g. Chairman, Counsellor [ ] Others specify………..

6b. If you are a traditional birth attendant what do you specialize in? Helping women to give birth [ ] Postpartum haemorrhage [ ] Prenatal care [ ] Postnatal care [ ] Abortion [ ] Child care Others specify………………

6c. Where did you learn to be a traditional birth attendant? Media Radio [ ] Media TV [ ] Relative [ ] (please specify e.g. uncle) Parents [ ] (please specify e.g. father) Nursing school [ ] Herbalist [ ] Others please specify…………………

6d. Have you ever lost anyone when helping them to give birth?

Yes [ ] No [ ]

6e. If YES, what caused her death?

1.

2.

3.

4.

5.

7a. Do you or any of your family members use plants to treat postpartum haemmorhage? Yes [ ] No [ ]

7b. If Yes, how many plants do you use/know?

| **Plant name** | **Habit** | **Plant part** | **Preparation method** | **Administration method** | **Dosage** | **Times in a day** | **Time after pregnancy** |
| --- | --- | --- | --- | --- | --- | --- | --- |
|  |  |  |  |  |  |  |  |
|  |  |  |  |  |  |  |  |
|  |  |  |  |  |  |  |  |
|  |  |  |  |  |  |  |  |
|  |  |  |  |  |  |  |  |

1. Habit = (1) Tree, (2) Shrub, (3) Herb, (4) Climber, (5) Creeper, (6) Sedge, (7) Grass, (8) Twinner
2. Part used = (1) Leaves, (2) Flowers, (3) Seeds, (4) Fruits, (5) Stem, (6) Stem bark (7) Stem wood, (8) Root, (9) Root bark, (10) Root wood, (11) Resin, (12) Hanging roots (e,g. Mutuba), (13) Tuber, (14) Whole
3. Preparation = (1) I give fresh plant material, (2) I squeeze direct into the mouth, (3) I cook the plant= decoction, (4) I soak in water=cold soaking, (5) I put in hot water =infusion, (6) I process a powder and add it to hot water, (7) I process a powder and add it to cold water, (8) I process a powder and add it to feed, (9) I process powder and boil, (10) I squeeze in water, (11) I heat the plant material and squeeze the liquid
4. Administer = (1) Oral, (2) Topical, (3) Rectal, (4) Parenteral

8a. Do you or any of your family members use plants to cause uterine contractions? Yes [ ] No [ ]

8b. If Yes, how many plants do you use/know?

| **Plant name** | **Habit** | **Plant part** | **Preparation method** | **Administration method** | **Dosage** | **Times in a day** | **Time during pregnancy** |
| --- | --- | --- | --- | --- | --- | --- | --- |
|  |  |  |  |  |  |  |  |
|  |  |  |  |  |  |  |  |
|  |  |  |  |  |  |  |  |
|  |  |  |  |  |  |  |  |
|  |  |  |  |  |  |  |  |

1. Habit = (1) Tree, (2) Shrub, (3) Herb, (4) Climber, (5) Creeper, (6) Sedge, (7) Grass, (8) Twinner
2. Part used = (1) Leaves, (2) Flowers, (3) Seeds, (4) Fruits, (5) Stem, (6) Stem bark (7) Stem wood, (8) Root, (9) Root bark, (10) Root wood, (11) Resin, (12) Hanging roots (e,g. Mutuba), (13) Tuber, (14) Whole
3. Preparation = (1) I give fresh plant material, (2) I squeeze direct into the mouth, (3) I cook the plant= decoction, (4) I soak in water=cold soaking, (5) I put in hot water =infusion, (6) I process a powder and add it to hot water, (7) I process a powder and add it to cold water, (8) I process a powder and add it to feed, (9) I process powder and boil, (10) I squeeze in water, (11) I heat the plant material and squeeze the liquid
4. Administer = (1) Oral, (2) Topical, (3) Rectal, (4) Parenteral

9a. Do you use plants for abortion? Yes [ ] No [ ]

9b. If Yes, how many plants do you use/know?

| **Plant name** | **Habit** | **Plant part** | **Preparation method** | **Administration method** | **Dosage** | **Times in a day** | **Time during pregnancy** |
| --- | --- | --- | --- | --- | --- | --- | --- |
|  |  |  |  |  |  |  |  |
|  |  |  |  |  |  |  |  |
|  |  |  |  |  |  |  |  |
|  |  |  |  |  |  |  |  |
|  |  |  |  |  |  |  |  |

1. Habit = (1) Tree, (2) Shrub, (3) Herb, (4) Climber, (5) Creeper, (6) Sedge, (7) Grass, (8) Twinner
2. Part used = (1) Leaves, (2) Flowers, (3) Seeds, (4) Fruits, (5) Stem, (6) Stem bark (7) Stem wood, (8) Root, (9) Root bark, (10) Root wood, (11) Resin, (12) Hanging roots (e,g. Mutuba), (13) Tuber, (14) Whole
3. Preparation = (1) I give fresh plant material, (2) I squeeze direct into the mouth, (3) I cook the plant=decoction, (4) I soak in water=cold soaking, (5) I put in hot water =infusion, (6) I process a powder and add it to hot water, (7) I process a powder and add it to cold water, (8) I process a powder and add it to feed, (9) I process powder and boil, (10) I squeeze in water, (11) I heat the plant material and squeeze the liquid
4. Administer =(1) Oral, (2)Topical, (3) Rectal, (4) Parenteral

10a. Are some of the uterotonic plants used in combination? Yes [ ] No [ ]

10b. How many combinations do you know?

One [ ] Two [ ] Three [ ] >3 [ ]

10c. If Yes, how many plants do you use/know when in combinations?

| **Plant name** | **Habit** | **Plant part** | **Preparation method** | **Administration method** | **Dosage** | **Times in a day** | **Time during pregnancy when an abortion takes place** |
| --- | --- | --- | --- | --- | --- | --- | --- |
| 1.  2.  3.  4. |  |  |  |  |  |  |  |
| 1.  2.  3.  4. |  |  |  |  |  |  |  |
| 1.  2.  3.  4. |  |  |  |  |  |  |  |
| 1  2  3  4 |  |  |  |  |  |  |  |
| 1  2  3  4  5 |  |  |  |  |  |  |  |

11a. When your uterus fails to contract, where do you seek treatment first? Pants alone [ ] Conventional drugs [ ] Both [ ]

11b. When you don’t recover the first time, where else do you seek treatment? Pants alone [ ] Conventional chicken drugs [ ] Both [ ]

12. How did you learn about uterotonic plants? Media Radio [ ] Media TV [ ] Relative [ ] (please specify e.g. uncle) Parents [ ] (please specify e.g. father) Veterinary doctor [ ] Herbalist [ ] Others please specify………………….

13. Where do you collect uterotonic plants from? Garden [ ] Compound [ ] Forest [ ] Pasture or grazing land [ ] Others specify .……………….

14. Do you think plants for contracting the uterus work? Very Effective [ ] Effective [ ] Not effective [ ]

15. How do you preserve uterotonic herbal remedies? Air-drying [ ] sun-drying [ ] freezing [ ] fermentation [ ] smoking [ ] I squeeze in water and keep in a bottle [ ] Others specify

16. For how long do you store uterotonic herbal remedies? (In days)……………………………

17. What challenges have you faced when using uterotonic herbal remedies?

………………………………………………………………………………………………………………………………………………………………………………………………………………………………………………………………………………………………………………………………………………

18. What challenges have you faced when using conventional medicine for uterine contraction? ………………………………………………………………………………………………………………

………………………………………………………………………………………………………………

18a. Do you grow some of the uterotonic plants? Yes [ ] No [ ]

18. b If Yes which ones? ……………………………………………………………………………………………………………………………………………………………………………………………………………………………………………………………………………….

18c. If No why? ……………………………………………………………………………………………………………………………………………………………………………………………………………………………………………………………………………….

19.a. Do you buy some of the uterotonic plants from the market? Yes [ ] No [ ]

19b. If Yes which ones

………………………………………………………………………………………………………………………………………………………………………………………………………………………………………………………………………………..

19c. If No why? ………………………………………………………………………………………………………………………………………………………………………………………………………………………………

20. a. What percentage of women prefer traditional birth attendants to modern hospitals?

≤10% [ ] 50% [ ] ≥50% [ ]

b. Why do they prefer traditional birth attendants?

c. Why don’t they like going to the hospital?

d. Have you had a friend who died at a traditional birth attendant? YES [ ] NO [ ]

e If YES, how many have you had? 1-5 [ ] 5-10 [ ] >10
